# Evaluation of the Effect of Photodynamic Therapy on CAM-Grown Sarcomas

**DOI:** 10.3390/bioengineering10040464

**Published:** 2023-04-11

**Authors:** Maximilian Kerkhoff, Susanne Grunewald, Christiane Schaefer, Stefan K. Zöllner, Pauline Plaumann, Maike Busch, Nicole Dünker, Julia Ketzer, Josephine Kersting, Sebastian Bauer, Jendrik Hardes, Arne Streitbürger, Uta Dirksen, Wolfgang Hartmann, Wiebke K. Guder

**Affiliations:** 1 Pediatrics III, University Hospital Essen, West German Cancer Center, 45147 Essen, Germany; christiane.schaefer2@uk-essen.de (C.S.); stefan.zoellner@uk-essen.de (S.K.Z.); pauline.plaumann@uk-essen.de (P.P.); josephine.kersting@uk-essen.de (J.K.); uta.dirksen@uk-essen.de (U.D.); 2 German Cancer Consortium (DKTK), Essen/Düsseldorf, University Hospital Essen, 45147 Essen, Germany; susanne.grunewald@uk-essen.de (S.G.); maike.busch@uk-essen.de (M.B.); nicole.duenker@uk-essen.de (N.D.); julia.ketzer@uk-essen.de (J.K.); sebastian.bauer@uk-essen.de (S.B.); jendrik.hardes@uk-essen.de (J.H.); arne.streitbuerger@uk-essen.de (A.S.); wiebke.guder@uk-essen.de (W.K.G.); 3 Faculty of Medicine, University Duisburg-Essen, 45141 Essen, Germany; 4 West German Cancer Center, University Hospital Essen, 45147 Essen, Germany; 5 Department of Neuroanatomy, Institute for Anatomy II, University Hospital Essen, 45147 Essen, Germany; 6 Department of Orthopedic Oncology, University Hospital Essen, 45147 Essen, Germany; 7 Division of Translational Pathology, Gerhard-Domagk-Institute of Pathology, University Hospital Muenster, 48149 Muenster, Germany; wolfgang.hartmann@ukmuenster.de

**Keywords:** sarcoma, tumor fluorescence, photodynamic diagnostics, photodynamic therapy, chorion-allantois membrane model, CAM, cell-derived xenografts, 5-ALA, 5-aminolevulinic acid

## Abstract

Resection margin adequacy plays a critical role in the local control of sarcomas. Fluorescence-guided surgery has increased complete resection rates and local recurrence-free survival in several oncological disciplines. The purpose of this study was to determine whether sarcomas exhibit sufficient tumor fluorescence (photodynamic diagnosis (PDD)) after administration of 5-aminolevulinic acid (5-ALA) and whether photodynamic therapy (PDT) has an impact on tumor vitality in vivo. Sixteen primary cell cultures were derived from patient samples of 12 different sarcoma subtypes and transplanted onto the chorio-allantoic membrane (CAM) of chick embryos to generate 3-dimensional cell-derived xenografts (CDXs). After treatment with 5-ALA, the CDXs were incubated for another 4 h. Subsequently accumulated protoporphyrin IX (PPIX) was excited by blue light and the intensity of tumor fluorescence was analyzed. A subset of CDXs was exposed to red light and morphological changes of both CAMs and tumors were documented. Twenty-four hours after PDT, the tumors were excised and examined histologically. High rates of cell-derived engraftments on the CAM were achieved in all sarcoma subtypes and an intense PPIX fluorescence was observed. PDT of CDXs resulted in a disruption of tumor-feeding vessels and 52.4% of CDXs presented as regressive after PDT treatment, whereas control CDXs remained vital in all cases. Therefore, 5-ALA mediated PDD and PDT appear to be promising tools in defining sarcoma resection margins (PDD) and adjuvant treatment of the tumor bed (PDT).

## 1. Introduction

Most malignant tumors that are identified as sarcomas have the potential for hematogenous metastatic spread [1]. Recurrent disease is associated with a dismal prognosis for sarcoma patients [2,3,4,5]. One of the main pillars of sarcoma treatment is wide-margin tumor resection [6,7]. Most importantly, it is not the width but the quality of the resection margin that has prognostic value [8]. Possible reasons for the formation of local soft tissue recurrences following tumor resection with histopathologically confirmed clear margins are: (1) undetected, small tumor thrombi within the micro-vessels surrounding the primary tumor or (2) discontinuous tumor cell clusters outside the tumor capsule due to tumor volume reduction after neoadjuvant therapy. 

When planning a tumor resection, a proper preoperative tumor staging is critical. Imaging techniques include radiographs, bone scintigraphy, MRI and (PET-) CT/MRI scans and build the foundation for tumor margin determination [7,9,10]. However, none of these current imaging techniques enable the depiction of microscopic tumor cell clusters distant from the main tumor. As a result, and in order to increase local control rates, adjuvant radiotherapy of the tumor bed is routinely recommended [11,12]. By facilitating the detection and eradication of microscopic lesions, fluorescence-based approaches may be able to improve resection margin adequacy. This would effectively reduce postoperative radiation field size as well as the adverse side effects caused by adjuvant radiotherapy. A proof-of-concept study showed that fluorescence-guided surgery of RFP-labeled osteosarcomas led to improved disease-free survival compared to bright-light surgery in a murine model [13]. Furthermore, a sarcoma case series that implemented near-infrared fluorescence-guided surgery revealed a five-fold decrease in the frequency of unexpected positive margins [14]. 

Photodynamic diagnosis (PDD) has become a valuable fluorescence-based tool in various medical disciplines, including surgical oncology [15,16]. For example, PDD with 5-aminolevulinc acid (5-ALA) increased the resection rate of gliomas from 40% to 80%, and the total-removal rate of glioblastomas by resection surgery improved from 36% to 66% [17]. Furthermore, 5-ALA-induced PDD has been shown to be a safe and valid tool even after chemotherapy and radiotherapy prior to resection of brain tumors [18,19]. 5-ALA is a natural substrate in heme-biosynthesis, and its application in fluorescence-guided surgery has been shown to be safe [18,20]. 5-ALA is non-targeted and non-selective for tumor cells. It is metabolized into heme in normal cells. However, because of the low activity of the enzyme ferro-chelatase in cancer cells, the heme-precursor protoporphyrin IX (PPIX) accumulates and can subsequently be excited by blue light, inducing red light emission. Upon excitation with red light, PPIX produces reactive oxygen species that lead to apoptosis and/or necrosis of the affected cells [21]. This process is described as photodynamic therapy (PDT). 

A direct impact of PDT on the cell viability of myxofibrosarcoma and osteosarcoma has been demonstrated in vitro [22,23]. However, previous studies reported that tumors are destroyed largely by hypoxia exposure that occurs for several hours to days as the result of tumor-feeding vessel disruption, rather than by direct tumor cell toxicity [24,25,26]. A locally restricted inflammatory response that can lead to nonspecific destruction of tumor cells may play an additional role [27]. We hypothesize that PDD and PDT may improve the quality of resection margins in sarcoma surgery. 

The chorio-allantoic membrane (CAM) of fertilized chick eggs is an in vivo model that is the ideal substrate for tumor xenografts due to its strong vascularization and natural immunodeficiency [28,29]. A previous study investigated the impact of 5-ALA-induced PDT on tumor vitality of patient-derived tumor xenografts (PDXs) grown on the CAM. However, a high rate of spontaneous tumor regression caused by insufficient perfusion of CAM-distant portions of the PDXs led to inconclusive results [30]. To reduce the risk of spontaneous regression in this study, further evaluation of the effects of PDT on sarcomas was performed on the cell-derived xenografts (CDXs) of several sarcoma subtypes. The main questions were as follows: (1) Is CAM grafting feasible, and what are the success rates? (2) Do these engraftments show PPIX fluorescence? (3) Is the fluorescence intensity higher and more uniform in CDXs than in PDXs? Finally, (4) Can a therapeutic impact of PDT on CAM-grown CDX tumors be determined? 

## 2. Materials and Methods

### 2.1. Patient Characteristics

Tumor material from 15 patients consisting of 12 different sarcoma subtypes with localized, recurrent and metastasized disease states and any prior treatments were included. Median patient age was 34 (19–71) years at the time of sample collection, with a proportion of 53.3% male (median age: 51 (20–71)) and 46.7% female subjects (median age: 20 (19–66)). The extent of pretreatment ranged from no prior treatment before sample collection (for example, cell lines H7L6, H7N5 and E0R4) to intensely pretreated (for example, cell lines I0A0 and mLPS-SN1; Table 1).

### 2.2. Sample Preparation of Tumor Tissue

An experienced sarcoma reference pathologist analyzed patient material immediately after surgery. If the obtained tumor tissue amount was sufficient for a reliable diagnosis, the pathologist separated a fresh tumor tissue sample. Tumor tissues were dissolved for cell culturing prior to inoculation on the CAM. One exception was H7N5, which was applied onto the CAM as both tissue pieces (PDXs) and cell engraftments (CDXs) for direct comparison of both methods. In the case of PDXs, tumor tissue was cut into cubes of about 1 mm^3^ which were kept in a droplet of DMEM (Gibco™ Dulbecco’s Modified Eagle Medium, high-glucose, GlutaMAX™) to prevent the tissue from drying out before engraftment. 

### 2.3. Primary Cell Culture Preparation

Tumor tissues were minced with a scalpel into cubes about 1 mm^3^ in size. These were transferred into a 50 mL centrifuge tube containing 5 mL trypsin (trypsin EDTA, 0.25%). The mixture was incubated at 37 °C for 30–45 min with constant shaking (300 revolutions per minute (rpm)) until the trypsin turned opaque. The suspension was strained through a sieve (MACS^®^ SmartStrainers 100 µm) into a 15 mL centrifuge tube. Trypsin was inhibited by the addition of 7 mL DMEM supplemented with 10% fetal bovine serum (FBS), and the suspension was centrifuged for 5 min at 300 g. The supernatant was discarded. The cell pellet was resuspended in 7 mL DMEM + 10% FBS, and the suspension was transferred into a collagen-coated T25 tissue culture flask. The medium was supplemented with 1% penicillin/streptomycin for the first two to three media changes. 

### 2.4. Short-Term Cell Cultures

After primary cell cultures reached a confluency of about 80–90%, cultures were split (1:2; split factors were later adapted to proliferation rates) and expanded into multiple T25 or T75 tissue culture flasks. Before transplanting cells onto the CAM, a T75 flask had to reach at least 70% confluency to obtain enough cells for multiple engraftments. Not all attempts to culture patient material were successful, and about half of the cell cultures used in this study stopped dividing prior to the 10th passage. The cell lines EW-7 and TC-32 were obtained commercially and were authenticated by STR profiling. All cell lines were free from mycoplasma infection, verified by PCR. 

### 2.5. CAM Model and 5-ALA Treatment 

The detailed protocol for CAM experiments was described previously [30]. Fertilized chick eggs were incubated at 37.8 °C in 60% humidity with 12 revolutions per day. On day 2 of embryonic development (EDD2), a 1.5–2 cm Ø window was cut into the eggshell and resealed using silk tape (3M™ Durapore™). For the remainder of the incubation, the eggs were set in a fixed position with the window facing upwards. On EDD9, the tape was removed, a vessel of the CAM was perforated with a scalpel and either 100 µL of cell suspension (1 × 10^6^ cells resuspended in 100 µL Corning^®^ Matrigel^®^ Matrix) or a 1 mm^3^ cube of tumor tissue was placed onto the perforated site. The window was then resealed. On EDD15, 200 µL of 10 mg/mL of 5-ALA was applied topically to the CAM and the eggs were incubated for another 4 h at 37.8 °C prior to PPIX fluorescence observation. The 4 h of incubation were chosen based on good fluorescence results in a former study [30]. It is important to note that the eggs used in this study were not specified as pathogen-free (SPF) and the working steps were not performed in a sterile environment. After ruling out all other potential sources of observed contamination, the incubator was deemed a contamination risk. Therefore, the inside of the incubator was disinfected by Terralin^®^ and the water for humidification was supplemented with AppliClear-water by AppliChem GmbH for germ reduction. 

### 2.6. Photodynamic Diagnosis: PPIX Fluorescence Detection

For PPIX fluorescence detection, the eggs were placed into a dark chamber with a pinhole in its top. Images of the tumors were taken using a PDD-adjusted endoscope (System blue compounds PENDUAL blue HD Camera Head, PANOVIEW blue Telescopes and ENDOLIGHT LED blue by Richard Wolf GmbH) that was inserted through the pinhole. For each tumor, images at both white light and blue light (405 ± 10 nm) excitation were taken. 

### 2.7. Photodynamic Therapy: Red Light Excitation of Tumors

After PPIX fluorescence documentation, a subgroup of eggs was treated by exposure to red light (635 ± 10 nm; 10 J/cm^2^; 26/31/36/43/50 mW/cm^2^) at a distance of 15 cm using a MultiLite^®^ Daylight PDT Lamp (GME German Medical Engineering GmbH, Dreikönigstr. 6-8, 91054 Erlangen, Germany). PDT-treated and untreated eggs were incubated for another 24 h at 37.8 °C prior to a second photo documentation, followed by termination and tumor harvest. 

### 2.8. Histological Evaluation of Tumor Viability and Regression

Harvested tumors were fixated in 3.7% buffered formalin. Dehydration, paraffin embedding and hematoxylin and eosin (H&E) staining were performed following standard protocols. Tumor viability/regression was assessed as stated by Guder et al. [30]. Proportions of vital, necrotic and fibrotic tissue were determined semi-quantitatively. CDX tumors were categorized as viable (>75% vital tissue), partially regressive (>50% vital tissue) or regressive (<50% vital tissue). 

### 2.9. Fluorescence Intensity Measurements

PPIX fluorescence intensities were classified using two different approaches. Firstly, the fold change in mean grey value of tumors against the background signal of the CAM was calculated. For this, a region of interest (ROI) containing the tumor was created by the freehand selection tool and its mean grey value was measured in ImageJ (Version 1.53c; Java 1.8.0_172 (64-bit)). Next, the selection was enlarged by 200 pixels (which was about one tumor diameter) in all directions, the tumor-containing ROI was deleted from the selection and its mean grey value was measured. The mean grey value of the tumor was divided by the mean grey value of the surrounding CAM tissue to calculate the fold change in mean grey value. 

Secondly, tumors were categorized as non- (0), weakly (1), intermediately (2) and strongly fluorescent (3) by subjective assessment with a focus on peak intensity tumor sites. In a blind experiment, two researchers independently assessed the fluorescence of the respective tumor entity while performing PDD. In the event of differing fluorescence assessment, the mean of both values was taken. The values were treated as continuous variables to calculate average fluorescence scores.

The correlation of both fluorescence intensity measurements was tested using a linear regression model (analysis and graphing software GraphPad Prism^®^ version 9.5.1, Boston, MA, USA). 

### 2.10. Analysis of PPIX Fluorescence Intensity Changes after PDT

Images of the PPIX fluorescence were taken immediately before and one hour after PDT treatment. The fold change of tumor grey value versus the background grey value was calculated for both time points and tested for significant changes between the two time points by a paired student’s *t*-test with GraphPad Prism^®^. 

## 3. Results

### 3.1. Chorio-Allantoic Membrane (CAM) Model

The survival rates of the chick embryos ranged from 50 to 80%, which is consistent with previous observations [31]. Unfertilized eggs accounted for about 3% of the total and were not taken into consideration. A total of 524 fertilized eggs were prepared, of which 376 (71.8%) survived until the time point of experiment termination. The greatest loss in eggs occurred within 3 days after opening the eggshell at EDD2. 

### 3.2. Tumor Models Derived from Patient Material on the CAM

In this study, 16 primary and short-term cultures were derived from 12 different sarcoma subtypes (15 patients). These subtypes comprised chondrosarcoma, chordoma, desmoplastic small round cell tumor (DSRCT), endometrial stromal sarcoma, Ewing sarcoma (EwS), giant cell tumor of bone (GCTB), gastrointestinal stromal tumor (GIST), malignant peripheral nerve sheath tumor (MPNST), myxofibrosarcoma, myxoid liposarcoma, osteosarcoma and synovial sarcoma (Table 1). The inclusion of patient samples was determined by availability. We decided not to control the included patient samples for disease stage or prior treatments to be able to investigate and compare the applicability of 5-ALA in any number of clinically relevant settings. 

In total, 337 engraftments were generated on the CAM, including 23 PDXs and 314 CDXs. Two established EwS cell lines (EW-7 & TC-32) were the basis for 143 CDXs. The commercial cell lines were included to increase sample size, monitor their fluorescence intensity distribution and compare their behavior with the cell cultures freshly derived from patients in our experiments. One hundred and seventy-one CDXs were derived from primary and short-term cell cultures. A total of 46 grafts (13.6%) were lost due to embryo death, 42 grafts (12.5%) were lost due to contamination and 14 grafts (4.2%) did not result in a visible tumor. Thus, the overall success rate for generating evaluable xenografts on the CAM was 70.3% (Table 1). 

At the time of application onto the CAM, cell-Matrigel^®^ suspensions appeared as pinkish transparent droplets. After three days, solid white tumors were observed. Opacity correlated with cell density. In a subset of CDX grafts, low cell density and overrepresented extracellular matrix were observed and considered to be residual Matrigel^®^ in combination with a low cell proliferation rate. The mean tumor size was 4.96 ± 1.39 mm in the longest diameter and did not differ between established and primary cell line-derived xenografts (established: 4.87 ± 1.29 mm; primary: 5.03 ± 1.45 mm). 

### 3.3. Photodynamic Diagnostic (PDD)

**Figure 1:**
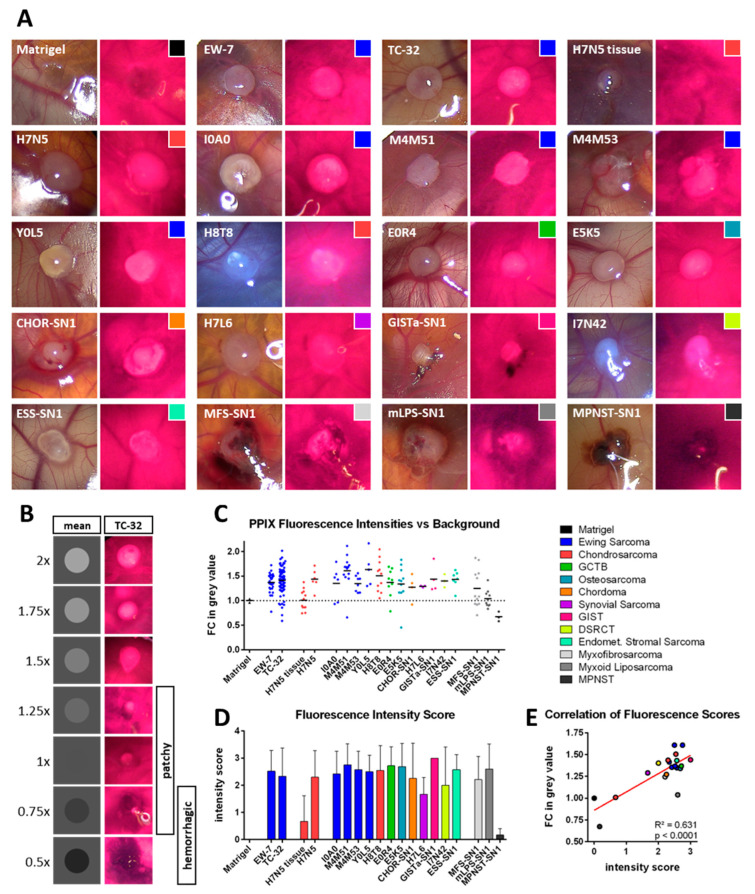
Photodynamic Diagnosis of Sarcoma xenografts on the chorio-allantoic membrane. (**A**) Overview of representative images of sarcoma xenografts upon white light (left images) and blue light (405 nm; right images) excitation. PPIX fluorescence presents as pinkish red. (**B**) For data visualization purposes, fold changes (FC) in mean grey values are depicted as grey circles on a grey background. TC-32 tumors with corresponding fluorescence intensities are shown adjacently. Tumors with an FC in mean grey value between 0.75 and 1.25 showed in most cases a patchy/partial fluorescence, while values below 1 were associated with a hemorrhagic phenotype. (**C**) FCs in mean grey value between tumors and their surrounding CAM tissue were calculated for all tested xenografts and cell-free Matrigel^®^ as a control. (**D**) A fluorescence intensity score was determined qualitatively for each tumor visually while performing PDD. Mean intensity scores with the respective standard deviation are depicted. (**E**) Correlation of mean values of both intensity scores from (**C**,**D**). The legend shows the color code for the different sarcoma subtypes and is shared by (**A**,**C**–**E**).

PPIX fluorescence was observed in CDX tumors of all tested sarcoma subtypes (Figure 1A). While there was no change in mean grey value of cell-free Matrigel^®^ droplets compared to the CAM surface, CDX tumors derived from established EwS cell lines showed increased fluorescence intensities. When comparing mean values, EW-7 tumors showed a 1.368-fold and TC-32 tumors a 1.415-fold increase in intensity over the background (Figure 1B,C). 

Direct comparison of PDX and CDX tumors from the same specimen (H7N5, chondrosarcoma) confirmed that CDX tumors exhibited more uniform PPIX fluorescence, whereas PDX tumors presented a patchier and darker phenotype. As a result, CDX tumors had higher mean intensities than PDX tumors (Figure 1C). 

The majority of sarcoma subtypes showed CDXs with an average fold change in intensity against the CAM background above 1.25. Though the CAM showed a strong background fluorescence, CDX fluorescence was distinguishable from the surrounding CAM tissue. The subjective fluorescence intensity scoring concentrated more on peak fluorescence and was assessed visually by two investigators. There were no major differences in the average fluorescence score between different tumor entities, suggesting that most tumors were easily distinguishable from the background tissue by their PPIX fluorescence (Figure 1C,D). The Matrigel^®^ control was classified as non-fluorescent (*n* = 2, 100%); CDXs from established cell cultures were classified as non-fluorescent in nine (10%), as weakly fluorescent in five (5.6%), as intermediately fluorescent in 17 (18.9%) and as strongly fluorescent in 59 cases (65.6%). CDXs from primary cell cultures (with the exception of the cell lines MFS-SN1, mLPS-SN1 and MPNST-SN1) were classified as non-fluorescent in five (6%), as weakly fluorescent in four (4.8%), as intermediately fluorescent in seven (8.4%) and as strongly fluorescent in 67 cases (80.7%).

Exceptions of good discernability between tumor and CAM tissue by fluorescence were the H7N5 tissue-derived xenotransplants and the MPNST-SN1 CDXs, which had low average subjective fluorescence intensity scores (Figure 1D). Furthermore, the CDXs from MFS-SN1, mLPS-SN1 and MPNST-SN1 had the least change in mean grey value (with an average fold change in intensity below 1.25). These subtypes had a strong tendency to hemorrhage (Figure 1A,C). The H7N5 PDXs were classified as non-fluorescent in seven (58.3%), as weakly fluorescent in three (25%), as intermediately fluorescent in one (8.3%) and as strongly fluorescent in one case (8.3%). The CDXs tending to hemorrhage (MFS-SN1, mLPS-SN1 and MPNST-SN1) were classified as non-fluorescent in four (16%), as weakly fluorescent in one (4%), as intermediately fluorescent in seven (28%) and as strongly fluorescent in 13 cases (52%). 

Overall, calculated fluorescence intensity measurements (semi-quantitative; Figure 1C) and subjective fluorescence intensity assessments (qualitative; Figure 1D) showed significant positive correlation (Figure 1E). Tumors with low cell density and high content of residual Matrigel^®^ fluoresced as intensely as dense tumor grafts (compare Figure 1C,D with Figure S1), indicating that even sparsely distributed tumor cells can reach high intensities in PPIX fluorescence. Unfortunately, we were unable to accurately measure tumors smaller than 1 mm^2^ because the CDX tumors were explanted under white light conditions and were lost due to their small size. The smallest fluorescent tumor we were able to measure had a surface area of 0.974 mm^2^.

**Figure 3:**
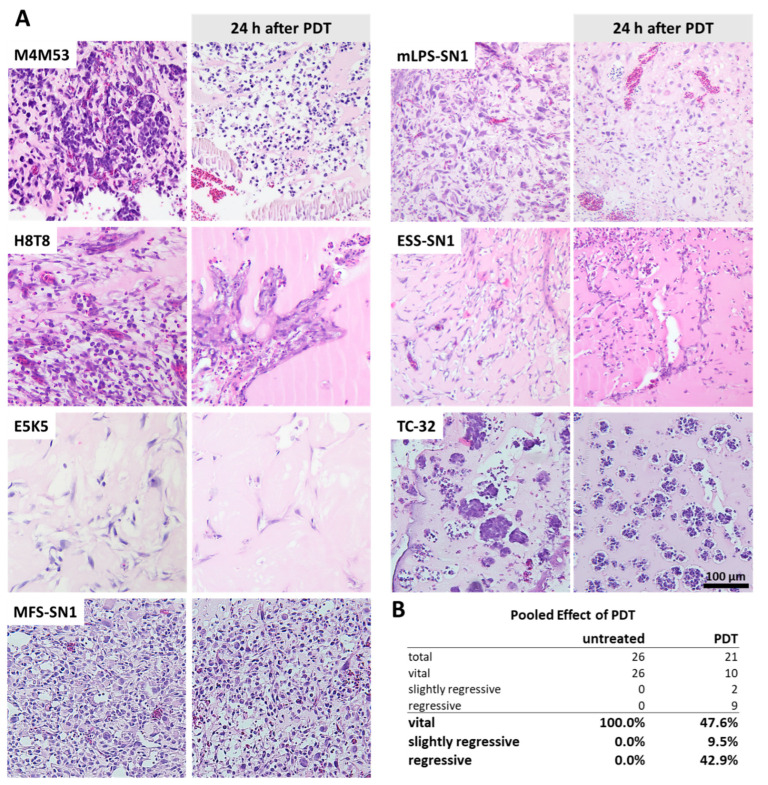
Histopathologic changes upon PDT treatment. (**A**) Histopathology of untreated and PDT-treated CDX tumors of different sarcoma subtypes. Tumors were harvested 24 h after PDT treatment. Ewing sarcoma: M4M53, H8T8, TC-32; myxoid liposarcoma: mLPS-SN1; endometrial stromal sarcoma: ESS-SN1; osteosarcoma: E5K5; myxofibrosarcoma: MFS-SN1. (**B**) Pooled effect of PDT on tumor regression. Details on composition (number of untreated/number of PDT treated): M4M53 (1/3); H8T8 (1/2); E5K5 (6/2); ESS-SN1 (4/3); mLPS-SN1 (3/4); MFS-SN1 (5/3); TC-32 (6/4).

The extent of pretreatment of the tumor sample showed no apparent negative effect on either cell proliferation rates or tumor fluorescence intensity. For example, the heavily pretreated EwS cell lines I0A0 and M4M53 neither showed lower cell density in the Matrigel^®^ (compare in Figure 3 and Figure S1) nor differed in their mean fluorescence intensities from other less pretreated cell lines (compare Figure 1C,D). However, the analysis of putative pretreatment effects is limited to descriptive analysis in this study for a lack of good controls. 

### 3.4. Photodynamic Therapy (PDT)

To identify the PDT dose that was best tolerated by the chick embryos, we determined survival rates of embryos following 10, 20 and 30 J/cm^2^ of red-light exposure (635 nm). While all untreated eggs survived, decreasing amounts of eggs survived with increasing PDT doses (Table 2). We accepted an approximate 33% egg loss and proceeded with 10 J/cm^2^ in subsequent experiments to avoid a second-order error.

CDX tumors of TC-32, H8T8, M4M53, E5K5, ESS-SN1, mLPS-SN1 and MFS-SN1 were treated with PDT (the choice for these CDXs was based on availability). In total, 30 CDXs were treated with a constant dose of red light (10 J/cm^2^; 635 nm) at different intensities (26 mW/cm^2^, 31 mW/cm^2^, 36 mW/cm^2^, 43 mW/cm^2^ or 50 mW/cm^2^). Still, nine of the embryos died after treatment (higher intensities led to higher risk of death). Non-viable eggs and contaminated tumors were excluded from further analysis. 

**Figure 2:**
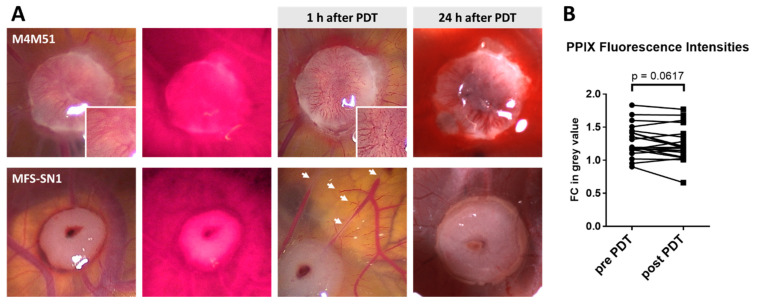
Macroscopic changes upon PDT treatment. (**A**) Examples of blood vessel fragmentation within (upper panel, compare zoom-ins before and after PDT) and surrounding (lower panel, indicated by white arrows) the tumor. Twenty-four hours after PDT, tumors presented as either hemorrhagic (upper panel) or ischemic (lower panel). (**B**) For a subgroup of tumors, PPIX fluorescence intensity was measured before (pre-PDT) and 1 h after PDT (post-PDT). The fold change (FC) in mean grey value of tumor against the background is depicted. Statistical analysis: paired Student’s *t*-test. M4M51 = Ewing sarcoma; MFS-SN1 = myxofibrosarcoma.

One hour after PDT treatment, tumors were documented and PPIX fluorescence of the tumors was assessed again. Blood vessels within and surrounding tumors were fragmented in all PDT-treated eggs, and some of the CAMs showed hemorrhages at the tumor site (Figure 2A). However, there was no significant decrease in fluorescence intensity (Figure 2B). Twenty-four hours after PDT treatment, most tumor sites presented as either ischemic (severe: 33%; mild: 23%), hemorrhagic (severe: 7%; mild: 7%) or showed a combination of both (20%; Figure 2A). Of all the tumor sites, 10% showed no distinct macroscopic effect of PDT on the CAM vessels. 

Upon termination of the experiment and tumor harvest, 21 PDT-treated tumors and 26 untreated tumors were analyzed histopathologically by H&E staining. Histopathological evaluation revealed tumor regression in a subset of the PDT-treated tumors (52.4%) while the untreated controls presented as vital in all cases (Figure 3A,B). An intensity-dependent increase in regression could not be observed due to the small sample size. 

## 4. Discussion

In this study, we were able to generate cell-derived engraftments of several sarcoma subtypes on the CAM with a success rate of 77.6%. PPIX fluorescence was observed in CDXs of all subtypes. In the employed technical setup, tumor fluorescence was distinguishable from the CAM background by image processing as well as visually, indicating that tumor fluorescence should be detectable in a surgical setting. With regard to its measurable background fluorescence of non-tumor tissue, the CAM seems to behave differently from healthy human tissue. Clinical evidence from brain tumor surgery performed after administration of 5-ALA, for instance, was not hampered by a significant level of background fluorescence from healthy brain tissue [17,18]. A possible cause for an increased rate of background fluorescence in the CAM model may be due to higher rates of pluripotency of embryonic tissues, when compared with more differentiated, healthy human tissue. Nevertheless, an increased knowledge of fluorescence properties of healthy tissues should be gained in future experiments. 

Hemorrhages severely impaired tumor fluorescence in this study. CDXs of the subtypes myxofibrosarcoma, myxoid liposarcoma and MPNST presented with increased tendencies to hemorrhage. These findings reflect known clinical behaviors of some sarcoma entities. Intratumoral hemorrhage has been reported for several soft tissue sarcomas and complicates tumor identification by MRI, as tumor lesions are easily mistaken for hematomas [32,33]. In direct comparison of chondrosarcoma models derived from the same patient sample, CDXs fluoresced more uniformly and with higher intensity than PDXs. Furthermore, control CDXs presented as vital in all cases (26/26 = 100%) in this study, whereas regression was observed in 16 out of 26 evaluable control PDXs (61.5%) in a previous study [30]. The high regression rate in PDXs was most likely caused by insufficient perfusion of CAM-distant PDX sites; hence, PPIX fluorescence was obstructed. While PDXs are tiny pieces of the original patient material resembling a wide spectrum of cell types within a tumor, CDXs are derived from primary and short-term cultures that underwent (few) passaging steps, creating a selective pressure towards higher proliferation rates. It has been shown that higher proliferation rates correlate with higher PPIX fluorescence in glioma [34]. Hence, the fluorescence intensities of CDXs might not be directly comparable to the in situ situation. However, even very sparse CDXs (with a low cell density within the Matrigel^®^) presented high fluorescence intensities (compare Figure 1, Figure 3 and Figure S1). In addition, tumors with a surface area smaller than 1 mm^2^ were detectable by their fluorescence, indicating that even tiny tumor residues are potentially observable in a clinical setting. Altogether, CDX models seem to be more suitable for the evaluation of PDD and PDT in the CAM system. 

The PDT treatment of CDXs led to observable therapeutic effects on the morphology of the surrounding tumor feeding vessels and on tumor viability in histopathological analysis. Rapid vessel fragmentation upon PDT exposure was followed by ischemia of the tumor in most cases in this study. PDT induced regression in about 50% of the CDXs while control tumors appeared viable in 100% of cases. The sample size was too small to observe an intensity-dependent increase of PDT effects. Furthermore, due to unequal tumor localization within the eggs (tumors engrafted in the outfield (corners of the eggshell) vs. infield (tumors directly below the center of the window)), red light excitation might have varied in its efficacy. 

Our experimental setup did not elucidate whether tumor regression upon PDT treatment was caused by a direct effect on tumor cells or an indirect effect by disrupting tumor-feeding vessels. According to the literature, the main cause of tumor cell destruction by PDT is the disruption of tumor-feeding vessels, which leads to hypoxia in tumor cells [24,25,26]. Unsurprisingly, an induction of neo-angiogenesis and anastomosis of vascular fragments upon PDT treatment has been observed, leading to re-perfusion of residual tumor cells. Thus, it is speculated that a combination of angiostatic drugs and PDT may lead to extended and more permanent periods of hypoxia with subsequent tumor cell death [35,36,37]. Future experiments with angiostatic treatments and spatially restricted excitation patterns could reveal whether the observed PDT effects on the tumors are direct, indirect or both. Interestingly, the primary and short-term EwS cultures showed behaviors similar to those of commercially available EwS cell lines. This suggests that commercial cell lines are a good model for further experiments that would require higher sample sizes. 

Though it has been shown to be safe [20], 5-ALA-based PDT had a lethal effect on some of the chick embryos at the employed excitation intensities. This effect is most probably the result of the CAM vessel destruction. As the CAM serves as a respiratory organ, the chick embryo heavily relies on its proper function [38]. Large areas of nonperfused CAM could have led to death by asphyxiation.

Considering that the effects of photodynamic therapy on tumors may be largely caused by vessel disruption, photodynamic therapy might appear unattractive for tumor sites with a proximity to vital vascular structures. However, studies in rabbits and rats observed that while high-dose PDT can lead to locally restricted alterations of nerves and blood vessels, it did not severely affect any vital organs or cause adverse clinical symptoms [39,40]. Further experiments are required to determine the PDT effects on healthy tissues surrounding the tumor bed. 

A major limitation of 5-ALA-induced PDT is the low penetration depth of red light (635 nm), which is limited to a few millimeters in tissue (0.2–4.0 mm in sarcomas) and consequently considered insufficient to target bulk tumors [41]. Therefore, adjuvant intraoperative PDT treatments to devitalize satellite tumor formations of the tumor bed after tumor resection may be an appropriate strategy. For instance, intraoperative PDT after the resection of neuroblastomas was able to reduce the recurrence rate in a murine model by half [42]. 

## 5. Conclusions

We feel strongly that this proof-of-principle study supports the likelihood that a combined PDD and PDT approach will improve the quality of tumor resection margins in sarcoma surgery both in primary and locally recurrent tumors. PDT may very well aid the destruction of micrometastases and tumor thrombi located in tumor-feeding vessels within the tumor bed that remain undetected by current imaging techniques. The main reason for tumor regression upon PDT treatment should be determined in further preclinical experiments to be able to increase the efficacy and ensure the safety of PDT treatment. As a first step towards a clinical administration of 5-ALA-induced PDD and PDT for musculoskeletal tumors, those treated by intralesional curettage may be feasible candidates (i.e., giant cell tumor of bone, atypical chondrogenic tumor). 

## Figures and Tables

**Table 1:** Sample characteristics. M4M51 and M4M53 were derived from different samples of the same patient. * Information taken from https://www.cellosaurus.org/search (accessed on 5 January 2023). † Histologically not typical for GCTB. ‡ Some of the samples were of poor quality for de-termining regression rates after PDT and were therefore not considered for analysis, which explains the delta compared to evaluable samples in PDD. n.a. = Not applicable; n.d. = Not defined.

ID	Entitity	Age	Sex	Grade	Sample Origin	Pretreatment	Inoculated Eggs	Loss (Egg Death)	Contaminated Eggs	No Visible Tumor	Evaluable Grafts (PDD)	Loss (Death after PDT)	Evaluable Grafts (PDT)	Confirmed Histology
CHOR-SN1	Chordoma	53	m	n.a.	resection, Os sacrum; metachronous lymphnodular, osseous and pulmonary metastases	radiotherapy, Imatinib, Sirolimus, Sorafenib, debulking	4	0	0	0	4	n.d.	n.d.	3
E0R4	GCTB	45	m	n.a.	curettage, proximal tibia	none	11	0	1	1	9	n.d.	n.d.	(7) ^†^
E5K5	Osteosarcoma	20	m	TNM: cT3 N0 M0 G high-grade	resection of primary site, proximal femur	2× Doxorubicin/Cisplatin, 3× MTX	12	1	0	0	11	2	8 ^‡^	10
ESS-SN1	Endometrial Stromal Sarcoma	19	f	TNM: T4 Nx M1 (OTH, PER) Stage: IV (UICC 2016 [8th Edition]) high-grade	palliative resection, middle and lower abdomen	3× Ifosfamid/Doxorubicin	10	0	6	0	7	0	7	7
GISTa-SN1	GIST	67	m	TNM (initial): pT4 cN0 cM0; current: Tx Nx M1 Stage (initial): IIIb (UICC 2016 [8th Edition]); current: Stage IV	resection of metastasis, right abdominal wall	laparotomy, BLU285	5	1	0	0	4	n.d.	n.d.	n.d.
H7L6	Synovialsarcoma	20	f	TNM: pT1 L0 V0 Pn0 R0 (UICC 2017 [8th Edition]) Stage: FNCLCC-Grading: 3 + 1 + 0 = 4 (G2)	resection, proximal upper arm	none	8	0	5	0	3	n.d.	n.d.	n.d.
H7N5	Chondrosarcoma	53	f	TNM: pT1 L0 V0 Pn0 R0 (UICC 2017 [8th Edition])	resection, lateral distal thigh	none	23 (PDXs)	5	6	0	12 (PDXs)	n.d.	n.d.	n.d.
							13 (CDXs)	2	2	3	6 (CDXs)	n.d.	n.d.	7
H8T8	Chondrosarcoma	48	m	TNM: ypT4 ypNX L0 V0 Pn0 R0 (UICC 2017 [8th Edition])	resection, dorsal thigh	3× Doxorubicin/Ifosfamide; radiotherapy	20	4	2	2	11	1	3 ^‡^	11
I0A0	Ewing Sarcoma	20	f	Stage: IV	metastasectomy of pulmonary metastasis	6× VIDE, 3× VAI; 4× Temoz./Irinot./Vinc., 1× ICE, 1× VAI; irradiation of the lung; 3× Temoz./Irinot.; 6× Topot./Cycloph.	13	1	5	1	6	n.d.	n.d.	5
I7N42	DSRCT	21	f	TNM: Tx N0 M1 (REN, PER) Stage: IV (UICC 2016 [8th Edition])	resection of pulmonary primarius	3× VIDE	4	0	0	1	3	n.d.	n.d.	1
M4M51	Ewing Sarcoma	22	m	TNM: M0	ascites fluid, recurrent tumor sites: neuroforamina and Os sacrum	6× VIDE, 8× VAI	20	3	4	0	13	n.d.	n.d.	9
M4M53						6× VIDE, 8× VAI; 4× Topotecan/Cyclophosphamide	8	0	0	0	8	2	4 ^‡^	6
MFS-SN1	Myxofibrosarcoma	71	m	TNM (initial): pT2b N0 M1 (PUL) G3 (MX) TNM (current): pT4, pN0(0/3), L0, V0, Pn0, R1, G high-grade	resection, right adductor approaching femur	resection	13	0	1	0	12	2	8 ^‡^	10
mLPS-SN1	Myxoid Liposarcoma	62	m	TNM: pT2a/b N0 M1 Stage: IV (UICC 2009 [7th Edition])	abdominal tumor debulking, Omentum and Colon Transversum	resection, 3× Doxorubicin/Ifosfamide; laparotomy, Ixoten, Trabectidin, Eribulin	10	0	0	0	10	2	7 ^‡^	9
MPNST-SN1	MPNST	66	f	TNM: rT0 N0 M1 pulm, per Stage: IV (UICC 2016 [8th Edition])	metastasis, small intestine section	radiotherapy	6	2	0	1	3	n.d.	n.d.	n.d.
Y0L5	Ewing Sarcoma	20	f	TNM: cT2 N0 M0 Stage: IIB (UICC 2016 [8th Edition])	resection, left femur	5× VDC, 4× IE; Denosumab	14	2	6	1	5	n.d.	n.d.	4
EW-7	Ewing Sarcoma	20 *	f *	n.a.	derived from metastatic site: Pleural effusion *	n.a.	44	13	3	0	28	n.d.	n.d.	5
TC-32	Ewing Sarcoma	17 *	f *	n.a.	derived from sampling site: Bone; left ilium *	n.a.	99	12	1	4	82	0	10	10

**Table 2:** Determination of suitable PDT dose in the CAM assay. Three different doses of red light (635 nm) at a fixed intensity (26 mW/cm^2^) were tested on embryos at EDD15 without xenografts. Following 24 h exposure, the viable embryos were counted and survivor percentage was calculated. To test for correlation between PDT dose and survivor percentage, the shown data was used to perform a linear regression analysis.

PDT Dose [J/cm^2^]	Viable Eggs [n]	Total [n]	Survivors [%]	Linear Regression Model
0	13	13	100.0	
10	5	8	62.5	m = −2.5 ± 0.4
20	3	8	37.5	R^2^ = 0.952
30	2	8	25.0	*p* = 0.024

## Data Availability

Not applicable.

## Supplementary Materials

The following supporting information can be downloaded at: https://www.mdpi.com/article/10.3390/bioengineering10040464/s1, Figure S1: Histopathology of CDX tumors.
