# Supplementary material for: Evaluation of the Effect of Photodynamic Therapy on CAM-Grown Sarcomas
Source: Bioengineering (Basel). 2023 Apr 11;10(4):464. doi: 10.3390/bioengineering10040464 (PMC10136229; doi:10.3390/bioengineering10040464)
Supplement: Supplementary file 1 [file bioengineering-10-00464-s001.zip › bioengineering-2203653-supplementary.pdf]

Article

Evaluation of the Effect of Photodynamic Therapy on CAM-Grown Sarcomas

Maximilian Kerkhoff [1,2,3,*], Susanne Grunewald [2,3,4], Christiane Schaefer [1,2,3], Stefan K. Zöllner [1,2,3], Pauline Plaumann [1,2,3], Maike Busch [2,3,5], Nicole Dünker [2,3,5], Julia Ketzer [2,3,4], Josephine Kersting [1,2,3], Sebastian Bauer [2,3,4], Jendrik Hardes [2,3,6], Arne Streitbürger [2,3,6], Uta Dirksen [1,2,3], Wolfgang Hartmann [7] and Wiebke K. Guder [2,3,6]

1. Pediatrics III, University Hospital Essen, West German Cancer Center, 45147 Essen, Germany; christiane.schaefer2@uk-essen.de (C.S.); stefan.zoellner@uk-essen.de (S.K.Z.); pauline.plaumann@uk-essen.de (P.P.); josephine.kersting@uk-essen.de (J.K.); uta.dirksen@uk-essen.de (U.D.)
2. German Cancer Consortium (DKTK), Essen/Düsseldorf, University Hospital Essen, 45147 Essen, Germany; susanne.grunewald@uk-essen.de (S.G.); maike.busch@uk-essen.de (M.B.); nicole.duenker@uk-essen.de (N.D.); julia.ketzer@uk-essen.de (J.K.); sebastian.bauer@uk-essen.de (S.B.); jendrik.hardes@uk-essen.de (J.H.); arne.streitbuerger@uk-essen.de (A.S.); wiebke.guder@uk-essen.de (W.K.G.)
3. University Duisburg-Essen, 45141 Essen, Germany
4. West German Cancer Center, University Hospital Essen, 45147 Essen, Germany
5. Department of Neuroanatomy, Institute for Anatomy II, University of Duisburg Essen, University Medicine Essen, Essen, Germany
6. Department of Orthopedic Oncology, University Hospital Essen, 45147 Essen, Germany
7. Division of Translational Pathology, Gerhard-Domagk-Institute of Pathology, University Hospital Muenster, 48149 Muenster, Germany; wolfgang.hartmann@ukmuenster.de

* Correspondence: maximilian.kerkhoff@uk-essen.de

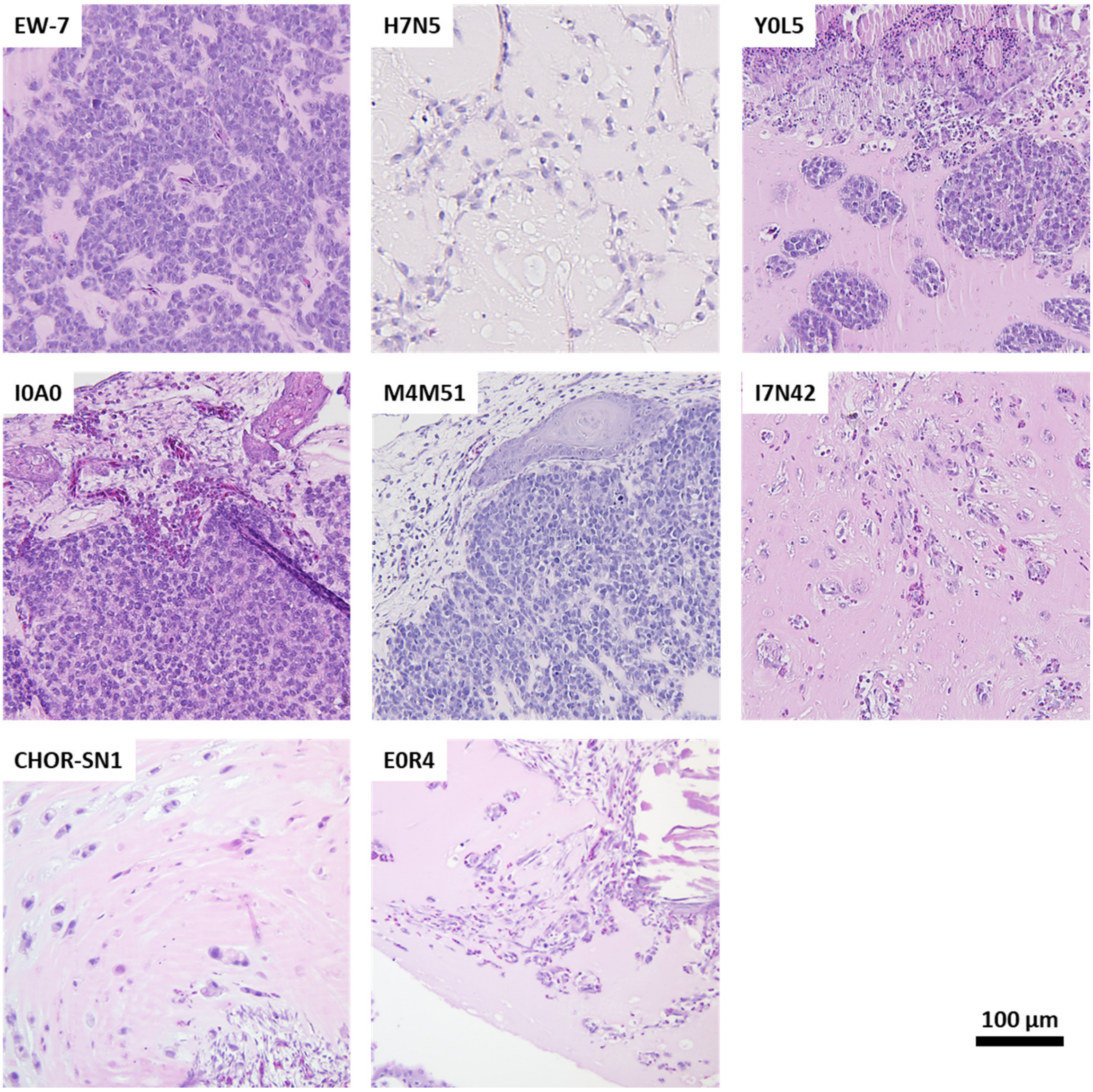

Supplementary Figure S1. Histopathology of CDX tumors. A) Histopathology of untreated CDX tumors of different sarcoma subtypes. Addition to Figure 3. Ewing Sarcoma: EW-7, Y0L5, I0A0, M4M51; Chondrosarcoma: H7N5; DSRCT: I7N42; Chordoma: CHOR-SN1; Giant Cell Tumor of Bone: E0R4.
